# Chemically directing d-block heterometallics to nanocrystal surfaces as molecular beacons of surface structure†
†Electronic supplementary information (ESI) available: GC-MS, XPS, EDS, EFTEM, FT-IR, XRD, ^1^H NMR, and ICP-OES data. See DOI: 10.1039/c5sc01474c


**DOI:** 10.1039/c5sc01474c

**Published:** 2015-07-28

**Authors:** Evelyn L. Rosen, Keith Gilmore, April M. Sawvel, Aaron T. Hammack, Sean E. Doris, Shaul Aloni, Virginia Altoe, Dennis Nordlund, Tsu-Chien Weng, Dimosthenis Sokaras, Bruce E. Cohen, Jeffrey J. Urban, D. Frank Ogletree, Delia J. Milliron, David Prendergast, Brett A. Helms

**Affiliations:** a The Molecular Foundry , Lawrence Berkeley National Laboratory , Berkeley , CA 94720 , USA . Email: bahelms@lbl.gov; b Department of Chemistry , University of California , Berkeley , CA 94720 , USA; c Stanford Synchrotron Radiation Lightsource , SLAC National Accelerator Laboratory , Menlo Park , CA 94025 , USA; d McKetta Department of Chemical Engineering , The University of Texas at Austin , Austin , Texas 78712 , USA

## Abstract

Our understanding of structure and bonding in nanoscale materials is incomplete without knowledge of their surface structure. Needed are better surveying capabilities responsive not only to different atoms at the surface, but also their respective coordination environments. We report here that d-block organometallics, when placed at nanocrystal surfaces through heterometallic bonds, serve as molecular beacons broadcasting local surface structure in atomic detail. This unique ability stems from their elemental specificity and the sensitivity of their d-orbital level alignment to local coordination environment, which can be assessed spectroscopically. Re-surfacing cadmium and lead chalcogenide nanocrystals with iron- or ruthenium-based molecular beacons is readily accomplished with trimethylsilylated cyclopentadienyl metal carbonyls. For PbSe nanocrystals with iron-based beacons, we show how core-level X-ray spectroscopies and DFT calculations enrich our understanding of both charge and atomic reorganization at the surface when beacons are bound.

## Abstract 2

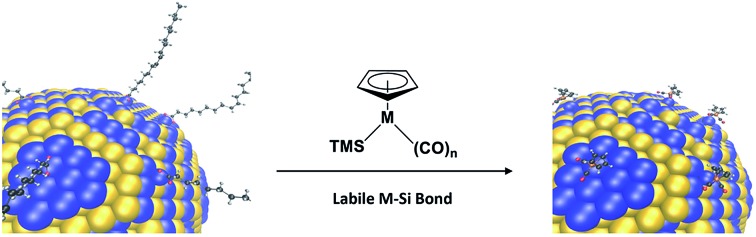
d-block heterometals reveal local surface structure.

## Introduction

Modern syntheses of colloidal semiconductor nanocrystals (NCs) yield dispersible materials sterically stabilized by organic ligands.^1^ The interface between a NC and its coordinating ligand sphere is widely recognized to influence the NC's physiochemical properties,^2^–^5^ mesoscale assembly,^6^–^8^ and even chemical reactivity.^9^–^11^ Elucidating its structure, which is both compositionally and topologically heterogeneous, is difficult even with leading-edge electron microscopy,^12^,^13^ scanning probe methods^14^ and spectroscopies.^15^–^17^ The experimental difficulty in resolving atomic details at buried organic–inorganic interfaces is further compounded by the spectroscopic insensitivity of atoms at that interface to differences in local coordination geometry. Additional complications arise when ligand motifs bonded to the NC surface (*e.g.*; through C, N, O, S, or P atoms) are present elsewhere in the system, either as part of the nanocrystal or as an adventitious impurity, which prevents definitive assignments of structure to specific spectral features. Given these limitations, only putative representations of the ligand–NC interface have been available to advance our understanding of NC behavior.

**Fig. 1:**
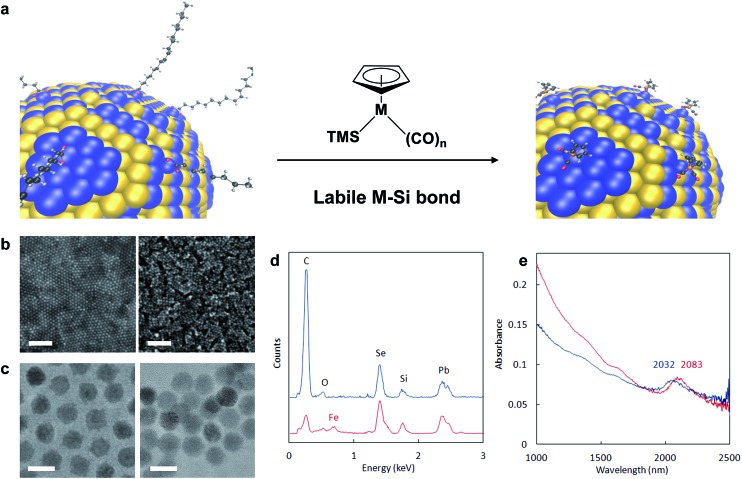
Reactive ligand exchange yielding semiconductor nanocrystals passivated with heterometallic molecular beacons (HMBs). (a) A PbSe surface representing 1/8^th^ of a 7 nm nanocrystal undergoes re-surfacing with trimethylsilylated cyclopentadienylmetal carbonyls, which feature a labile M–Si bond that aids in removing native coordinating ligands from the NC surface. Atoms are represented as: Pb = blue, Se = yellow, C = black, H = grey, O = red, Fe = orange. For M = Fe (Fp-TMS) or Ru (Rp-TMS), *n* = 2. TMS = trimethylsilyl. (b) SEM images of PbSe thin films on silicon substrates capped by native ligands (PbSe-OA, left) and after exchange to Fp beacons (PbSe-Fp, right). Scale bars are 100 nm. (c) TEM images of PbSe-OA (left) and PbSe-Fp (right). Scale bars are 10 nm. (d) EDS of NC films on silicon substrates before (PbSe-OA, blue) and after (PbSe-Fp, red) beacon binding. Spectra have been offset for clarity. (e) Diffuse reflectance of PbSe-OA (blue) and PbSe-Fp (red) films showing a red-shift in the first exciton peak after Fp binding to Pb(ii) at the NC surface.

We present here an alternative strategy, where conventional interfacing by main group elements at the NC surface is co-opted for one based on d-block organometallic complexes (Fig. 1). By design, these complexes feature transition elements that are distinct from any present in the NC, and are bonded to the NC surface through direct metal–metal bonds. Doing so deliberately couples the heterometal's d-orbitals to the nanocrystal's electronic states. Since the heterometal's L_2,3_ edges are extremely sensitive to both oxidation state and electronic interactions in their ligand field,^18^ their spectroscopic signatures as revealed by core-level X-ray techniques are thereby responsive to both charge and atomic re-organization at the interface. Using iron-based heterometallics as molecular beacons^19^ of PbSe surface structure, our analysis of their X-ray spectra using DFT calculations supports strong heterometal–NC coupling. For PbSe, we also noted an unexpected reorganization of surface Se when iron complexes were bound. Our work highlights the remarkable opportunities granted to these heterometallic molecular beacons (HMBs) in unraveling NC surface structure; this information is complementary to that gleaned by using vibrational reporters^15^ of surface composition and by using NMR reporters of ligand-surface stoichiometry and ligand-exchange dynamics.^17^ The unprecedented array of surveying functions conferred to HMBs may also lead to a new toolbox for manipulating NC properties.

## Experimental

### Materials and methods

Selenium (pellets, 99.999%), oleic acid, 1-octadecene (90%), lead(ii) oxide (99.999%), tri-*n*-octylphosphine (tech. grade 90%, Alpha Aesar), 1,2-hexadecanediol (90%), chlorotrimethylsilane (TMS-Cl), cyclopentadienyl iron(ii) dicarbonyl dimer, cyclopentadienyl ruthenium(ii) dicarbonyl dimer, potassium triethylborohydride (1.0 M in THF), potassium tri-*sec*-butylborohydride (1.0 M in THF), and trace metal grade (99.999%) nitric acid were purchased from Aldrich. Lead, selenium, cadmium, iron, and ruthenium standard solutions for inductively coupled plasma optical emission spectroscopy (ICP-OES) were purchased from Fluka. All chemicals were used as received. All manipulations, reactions, and characterization techniques were performed in a nitrogen glove box using dried and degassed solvents unless otherwise noted. Dicarbonyl(η^5^-cyclopentadienyl)(trimethylsilyl)iron (TMS-Fp) was synthesized according to slight modifications of a literature procedure^20^,^21^ (see below). Dicarbonyl(η^5^-cyclopentadienyl)(trimethylsilyl)ruthenium (TMS-Rp) and dicarbonyl(η^5^-cyclopentadienyl)(methyl)iron (Me-Fp) were synthesized according to literature procedures.^20^,^22^ CdSe,^23^ CdTe,^23^ CdSe/CdS dot-rods,^24^ and PbS truncated cuboctahedra^25^ were synthesized according to literature procedures. PbSe nanocrystals were synthesized as described below.

Scanning electron microscopy (SEM) images were recorded on a Zeiss Gemini Ultra-55 Analytical Scanning Electron Microscope using a beam energy of 5 kV and an In-Lens detector. An EDAX detector was used for EDS analysis. Transmission electron microscopy (TEM) was performed using a JEOL 2100F microscope equipped with Gatan's Tridiem imaging energy filter. The sample has been studied at beam energies of 120 and 200 kV. Energy-filtered TEM (EFTEM) images were obtained using a three-window method,^26^ where the background, calculated using two pre-edge windows, is subtracted from the image acquired past the edge energy. We used the Fe L_2,3_ edge at 706 eV and the S L edge at 163 eV, with spectrometer slit widths of 35 eV and 20 eV, respectively. The two images were then aligned based on image features and superimposed to visualize the relative distribution of the Fe and S atoms. ICP-OES was performed on a Varian 720 ES ICP optical emission spectrometer using an argon plasma. FT-IR spectra were obtained in air (unless otherwise noted) using a Perkin Elmer Spectrum One FT-IR Spectrometer. Photoluminescence spectra of CdSe–CdS and CdSe–CdS-Fp dot-rod NCs were recorded on a Horiba-Jobin Yvon Fluorolog II using 400 nm excitation. Samples were prepared by spin-coating NC films onto quartz slides; CdSe–CdS-Fp was prepared by ligand exchange on a spin-coated CdSe–CdS-oleate film. Samples were top-coated with a thin layer of cyclic olefin copolymer (Topas Advanced Polymers) to avoid contact with air. XRD was performed in air on a Bruker Gadds-8 diffractometer with a Cu-Kα source operating at 40 kV and 20 mA. Film thicknesses were determined using a Veeco Dektak 150 Surface Profilometer and confirmed with SEM cross-sectional imaging when possible (*i.e.* for films on silicon substrates). X-ray photoelectron spectroscopy (XPS) was performed under ultra-high vacuum conditions using a PHI 5400 XPS electron analyzer at 45° to the sample surface normal and a non-monochromatic Al X-ray source at 54.4° relative to the electron analyzer. Contributions to the XPS spectra from the Al-Kβ line have been filtered out during data processing. The area of collection was ∼1 mm^2^ on the sample surface.

Fe X-ray absorption spectroscopy (XAS) and X-ray emission spectroscopy (XES) were performed at beamline 7.0.1.1 at the Advanced Light Source at Lawrence Berkeley National Laboratories. The X-ray excitation area was ∼100 μm^2^ on the sample surface. XES were collected by a Scienta XES 300, a Nordgren-type, spherical grating, Rowland circle spectrometer using non-resonant excitation at 740 eV photon energy. XAS were collected by measuring the total fluorescence yield (TFY) and total electron yield (TEY) from the sample normalized against the photon flux (*I*_0_) during a scan of the X-ray monochromator. XAS measurements could be compared directly to control samples of unbonded Me-Fp, which were encapsulated in a cyclic olefin copolymer to avoid volatilization under UHV conditions. TEY measurements showed the same features as the TFY measurements for all samples. For PbSe nanocrystals the expected X-ray penetration and escape depth at the relevant photon energies is on the order of 100 nm, indicating that the spectra are composed from the emissive and absorptive character of atoms of the entire nanocrystal film. In this manner the character of atomic, site-specific binding environments for Fp beacons at the surface is measured for the full ensemble of available beacon-NC configurations.

Se K-edge XAS were collected at the Stanford Synchrotron Radiation Lightsource (SSRL) at beam line 6-2, experimental station 2, using a high-energy resolution crystal-based X-ray emission setup.^27^ A liquid nitrogen-cooled double-crystal monochromator Si(311) was used in addition to a Rh-coated parabolic focusing mirror to reject high harmonics. Samples were illuminated at 45-degree incident angle using an analysis area of 400 μm by 150 μm. Samples were handled and stored under argon prior to the measurements and a helium-filled container was used during measurements. The spectra were collected by monitoring the Se Lα_1_ emission line using a Bragg crystal analyzer with Si(844) orientation, providing a background-free spectrum. A silicon-drift diode was used to detect the monochromatized signals resolved from the Bragg analyzer. The data were normalized by fitting to polynomial and tabulated mass attenuation coefficients as described elsewhere.^28^

### Synthesis of oleate-passivated lead selenide (PbSe-OA)

Lead selenide nanocrystals with average diameter 7.2 nm ± 0.6 nm and first absorption feature at 2137 nm (in tetrachloroethylene) were synthesized following slightly modified standard procedures under an inert atmosphere.^29^,^30^ Briefly, a solution of Se (413 mg, 5.23 mmol) dissolved in tri-*n*-octylphosphine (13.2 mL, 0.4 M) was prepared in a 40 mL septum capped vial by stirring overnight in a nitrogen glovebox. 1,2-hexadecanediol (HDD, 688 mg, 2.66 mmol) was placed into a 50 mL three-necked flask, which was then purged with nitrogen. The solution of TOP-Se was injected into the flask containing the HDD and subsequently heated under vacuum at 120 °C for 1.5 h. In a 100 mL three-necked flask, lead(ii) oxide (589 mg, 2.64 mmol), oleic acid (2.5 mL), and 1-octadecene (11 mL) were heated at 120 °C for 1.5 h under vacuum to dry and degas the solution. Under nitrogen, the TOP-Se solution was cooled to 60 °C and the lead(ii) oleate solution was heated further to 150 °C. Once the temperature of the lead(ii) oleate solution had equilibrated, the thermostat was set to 130 °C and the TOP-Se was rapidly injected. After 1 h, the reaction was cooled in a water bath. The nanocrystals were then purified by precipitation three times from hexanes using ethanol, and two times with acetone.

### Synthesis of dicarbonyl(η^5^-cyclopentadienyl)(trimethylsilyl)iron (TMS-Fp)

A 100 mL Schlenk flask equipped with a stir bar was charged with cyclopentadienyl iron(ii) dicarbonyl dimer (1.06 g, 3.00 mmol) and THF (60 mL). The flask was sealed with a septum and removed from the nitrogen glovebox. Potassium triethylborohydride (9.00 mL, 1.0 M in THF) was added *via* syringe. The solution was stirred under nitrogen for 2 h at 40 °C. The solution was allowed to cool to ambient temperature before addition of TMS-Cl (1.52 mL, 12.0 mmol) *via* syringe. The solution was then allowed to stir for 2 h. The solvent was removed by evaporation under reduced pressure and subsequently the flask was brought back into a nitrogen glovebox. Hexanes (10 mL) were added and the mixture was filtered to remove salts into a clean 100 mL Schlenk flask. The hexanes filtrate was concentrated by evaporation under reduced pressure. The resulting oil was filtered from hexanes (5 mL) two additional times. The desired product was obtained as a brown-orange oil (572 mg, 2.79 mmol, 47% yield). The ^1^H NMR and FT-IR spectra were found to be in accord with literature values:^20^,^31^ ^1^H NMR (60 MHz, CDCl_3_): *δ* 4.64 (s, 5H), 0.39 (s, 9H) ppm. FT-IR (hexanes): *ν*(CO) = 1996, 1944 cm^–1^. Found: ^1^H NMR (500 MHz, CDCl_3_): *δ* 4.68 (s, 5H), 0.36 (s, 9H) ppm. FT-IR (CHCl_3_): *ν*(CO) = 1997, 1943 cm^–1^.

### General procedures for reactive ligand exchange reactions using TMS-Fp or TMS-Rp

Ordered nanocrystal films were prepared at a nitrogen-acetonitrile interface on single-side polished Si for SEM, EDS, XPS, XAS, and XES; random close packed films obtained by drop-casting were prepared on glass substrates for XRD and UV-vis-NIR absorbance. For ordered nanocrystal films, the substrate was immersed in ACN, a solution of PbSe-OA in hexanes was added carefully to the ACN to form a biphasic solution, the hexanes layer was allowed to evaporate, and then the nanocrystal film was deposited onto the underlying substrate by removal of the ACN *via* syringe. For re-surfacing with TMS-Mp reagents, the NC film was placed into a glass vial containing TMS-Mp (1 mL, 0.3 M in ACN) and allowed to soak for a desired amount of time (typically 30 min for TMS-Fp, and 1 h for TMS-Rp – during which ligand displacement was completed and Mp reached equilibrium saturation at the surface). The filtrate was removed *via* pipette. The film was then washed gently three times each with 800 μL portions of ACN and hexanes. Depending on the desired film thickness and continuity, multiple rounds of NC film deposition and treatment were performed.

### Preparation of ligand-stripped and Mp-passivated PbSe and CdSe for ICP-OES analysis

Ligand-stripped PbSe and CdSe samples were prepared and analyzed according to our previously reported procedures.^10^ For PbSe-Mp and CdSe-Mp (Mp = Fp or Rp), films were prepared on glass substrates as described above. Biphasic ligand exchange reactions were also investigated for comparison. Biphasic solutions of TMS-Mp (400 μL, 0.3 M in ACN) and PbSe-OA or CdSe-OA (5 mg mL^–1^ in hexanes, 400 μL) were prepared in centrifuge vials. After vortex mixing, the nanocrystals precipitated from solution. The filtrate was removed and the precipitated nanocrystals were washed with ACN and hexanes. After drying, the Mp-passivated nanocrystals were digested in HNO_3_ (70%, trace metal grade, 200 μL) and nanopure water (200 μL). The concentrations of Pb, Se, Cd, Fe, and Ru were determined using ICP-OES analysis of these samples against standard calibration curves. Each's surface chemistry was analyzed not only from multiple measurements of a single sample, but also by preparation of at least three independent samples.

The number of excess surface cations, *M*_surface_, and total number of cations in the NC, *M*_total_, were calculated for PbSe using the model developed by Wang and coworkers.^32^ The ratio of Pb_total_ : Se was taken from ICP-OES analysis with the known nanocrystal diameter of 7.4 nm to solve for the number of surface Pb atoms and the total number of atoms. For CdSe, the same model was applied using the bulk CdSe density *ρ*_CdSe_ = 5.816 and the covalent diameter of Cd as the thickness of the Cd shell *d*_Cd,shell_ = 0.288 with the known Cd : Se ratio from ICP-OES and known nanocrystal diameter of 3.6 nm. The PbSe model is an acceptable estimate for Cd_surface_ and Cd_total_ calculations for CdSe NCs, as both these compositions are non-stoichiometric, however, the number of *M*_surface_ for these two compositions may scale differently with size. More detailed modeling of CdSe would be required to confirm these estimated values.

As-synthesized, our PbSe and CdSe NCs exhibit cation-rich surfaces (10–25% surface excess of cationic metals, *M*_surface_, Table S1†). The number of coordinating ligands per *M*_surface_ atom have been rigorously determined for oleate-terminated Pb- and Cd-based semiconductor NCs : PbSe-OA and CdSe-OA exhibit OA : *M*_surface_ ratios of 1 : 1 and 2 : 1, respectively.^33^,^34^ The chemical identity of a second ligand at Pb_surface_ as might be necessary for charge balance is still unknown. Upon exchange of native oleates for various heterometallic beacons using TMS-Mp reagents, we were able to determine the extent of reaction and ensuing number of coordinating Mp beacons relative to *M*_surface_ quantitatively using inductively coupled plasma optical emission spectroscopy (ICP-OES) (Table S1†). Pristine PbSe-OA gave a Pb_total_ : Se ratio of 1.21 : 1.00 for NCs with average diameter of 7.2 nm, where Pb_total_ relates contributions from both Pb_surface_ and Pb_core_; Pb_surface_ is thus 21% of Pb_total_. Similarly, CdSe-OA was found to be terminated by Cd^2+^ with a surface excess in accord with previous literature reports (Cd_total_ : Se ratio of 1.11–1.23 : 1.00 for NCs with an average diameter of 3.6 nm). After treatment with Fp- or Rp-TMS both PbSe and CdSe retained essentially all of their excess surface cations. For both Fp-passivated PbSe and CdSe, measured Fp : *M*_surface_ ratios were consistent with those previously reported for oleates : PbSe–Fp gave a Fe : Pb_surface_ ratio of 0.95 : 1.00, while CdSe-Fp gave a Fe : Cd_surface_ ratio of 1.82 : 1.00. Extent of surface passivation with Rp under similar conditions was lower for both PbSe and CdSe, which could be due to the increased size of the metal center in addition to the decreased nucleophilicity of Rp^–^ relative to Fp^–^. The number of Mp beacons per NC was estimated from the percentage of Mp relative to *M*_surface_ times the number of *M*_surface_ atoms. The number of Mp beacons was divided by NC surface area to estimate the density of Mp beacons.

### Theoretical methods

All numerical calculations were computed at an atomistic level using density functional theory (DFT). Due to the large size of the ∼7 nm PbSe nanocrystals, which consist of several thousand atoms, we modeled the PbSe crystal as a periodically repeated 2-dimensional surface segment. A slab supercell exhibiting a 2 × 2 section of the {111} facet was constructed with ten layers in the surface-normal direction. Slabs were separated in the *z*-direction by vacuum thick enough to ensure no significant interactions between neighboring surfaces. Fp ligands were affixed to two of the four surface Pb atoms while OH groups were associated with the other two Pb surface atoms. The Fp ligands were arranged with alternating orientation due to steric constraints. The Se-terminated surface was passivated with hydrogen. The PbSe structure was initiated with the experimental lattice constant and the surface layers were allowed to reconstruct simultaneously with the relaxation of the ligand configuration.

DFT calculations were performed with the plane-wave based QuantumESPRESSO code.^35^ Atomic cores were modeled with ultrasoft psuedopotentials allowing calculations to be converged with a planewave cutoff of 40 Ry. Convergence in Brillouin zone integration was achieved with a 4 × 4 × 1 *k*-point sampling. The PBE exchange-correlation functional was used. All pseudopotentials used for ground-state calculations were taken from the QuantumESPRESSO pseudopotential repository. Specifically, we used Pb.pbe-d-van.UPF for Pb, Se.pbe-van.UPF for Se, Fe.pbe-sp-van.UPF for Fe, O.pbe-van_bm.UPF for O, C.pbe-van_bm.UPF for C, and H.pbe-van_bm.UPF for H. Certain calculations to model an X-ray excited final state used an alternate Fe pseudopotential which we constructed analogously to Fe.pbe-sp-van.UPF, but with a 2p hole.

## Results and discussion

### Re-surfacing metal chalcogenide nanocrystals with anionic cyclopentadienylmetal carbonyl complexes *via* chemical exchange

Our strategy to direct HMBs to semiconductor NC surfaces is based on a new chemoselective reaction between trimethylsilylated (TMS) cyclopentadienyl metal carbonyls^36^ (Mp) and native oxoanionic coordinating ligands bound to the NC's surface excess of metal cations (Fig. 1a). Highly polarized and chemically labile TMS-metal bonds enable transfer of electropositive TMS groups to oxoanionic ligands at NC surfaces, which renders native ligands non-coordinating.^37^,^38^ Upon elimination of native ligands as trimethylsilyl esters (Fig. S1†), open coordination sites generated at the NC surface are then substituted by anionic Mp molecular beacons. The driving force for coordination depends on NC composition and Mp nucleophilicity, which is known for many Mp anions.^39^ Anionic cyclopentadienyl iron and ruthenium dicarbonyls (Fp^–^ and Rp^–^, respectively) are highly nucleophilic complexes, and their TMS-derivatives were readily prepared^20^,^40^ and successfully applied to NC re-surfacing with HMBs.

Detailed analysis of NC re-surfacing with Fp beacons is shown for oleate-passivated 7.2 ± 0.6 nm PbSe NCs (Fig. 1b–e). Commensurate with native ligand exchange by Fp, interparticle spacing decreased from 2.9 nm to 1.3 nm (Fig. 1c). Energy dispersive X-ray spectroscopy (EDS) of PbSe-Fp films indicated a reduction in carbon content after ligand exchange and was accompanied by a new iron peak (Fig. 1d); the presence of Fe was also supported by X-ray photoelectron spectroscopy (XPS) (Fig. S2†). Re-surfaced NCs still exhibited quantum confinement: for PbSe, a 51 nm red shift after treatment with TMS-Fp was noted, consistent with increased electronic coupling and a change in effective dielectric constant in the film^10^,^11^ (Fig. 1e).

**Fig. 2:**
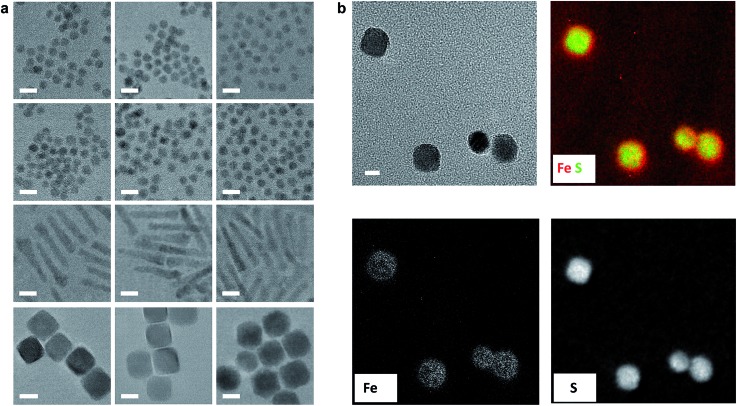
Generality of approach for various HMBs and NC compositions. (a) TEM images of semiconductor nanocrystals capped with native ligands (left column), Fp (middle column) or Rp (right column) molecular beacons. For corresponding EDS spectra, see ESI.† From top to bottom row: CdSe, CdTe, CdSe–CdS dot/rods, PbS truncated cuboctahedra. Scale bars are 10 nm. (b) EFTEM mapping of Fe and S for PbS-Fp. The bright field view is shown on the top left; scale bar is 10 nm. Overlaid false-color Fe and S L-edge maps are shown on the top right, supporting concentrated Fe atoms at the {100} faces of the cubes. The bottom images show the Fe and S maps before superposition.

Re-surfacing was demonstrated for oleate- and alkylphosphonate-passivated NCs of different sizes, shapes and compositions using either Fp- or Rp-TMS reagents (Fig. 2a & S3–S6†). Confirmation that Mp complexes were localized to and homogeneously distributed over NC surfaces was revealed by energy filtered TEM (EFTEM): for PbS-Fp truncated cuboctahedra, maps of the Fe L_2,3_ and S L-edges showed strong co-localization and registry with brightfield images (Fig. 2b), as well as a halo effect due to the higher number of Fp beacons projected along the edges; similarly, Fp beacons were localized to spherical CdSe surfaces (Fig. S7†).

The unique metal atoms present in HMBs enabled direct analysis of stoichiometry between excess NC surface atoms and Mp by inductively coupled plasma optical emission spectroscopy (ICP-OES) (see ESI†). Both PbSe and CdSe retained essentially all of their excess surface cations^2^,^3^ after treatment with Fp- or Rp-TMS (Table S1†). PbSe-Fp gave a Fe : Pb_surface_ ratio of 0.95 : 1.00, while CdSe-Fp gave a Fe : Cd_surface_ ratio of 1.82 : 1.00. The extent of surface binding by Rp under similar conditions was lower for both PbSe and CdSe, likely due to its larger size and lower nucleophilicity compared to Fp.

It is well known that surface constituents strongly influence the photoluminescence (PL) of colloidal nanomaterials.^41^–^44^ Indeed, CdSe–CdS dot/rods bearing Fp HMBs showed nearly complete PL quenching in thin films (Fig. S8†), suggesting trap states at the surface are prevalent after re-surfacing.

### Identification of the orbitals involved in HMB bonding to the nanocrystal surface

**Fig. 3:**
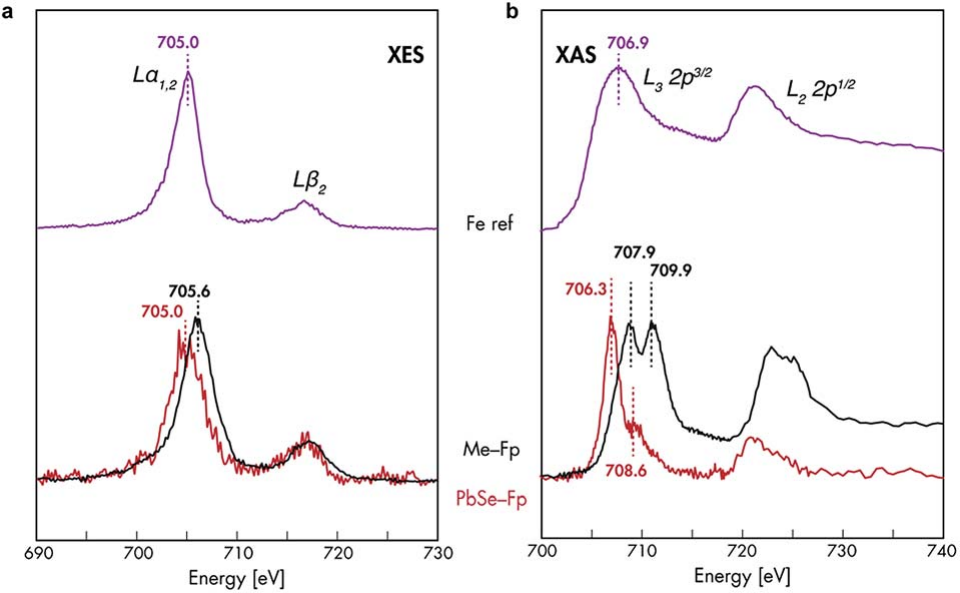
Fe XES and XAS analysis of bonding in PbSe-Fp. (a) Fe L-edge XES and (b) Fe L-edge XAS spectra for PbSe-Fp (red), with related compound Me-Fp (black), and Fe reference standard (purple) shown for comparison. Fe reference spectra have been vertically offset for comparison.

**Fig. 4:**
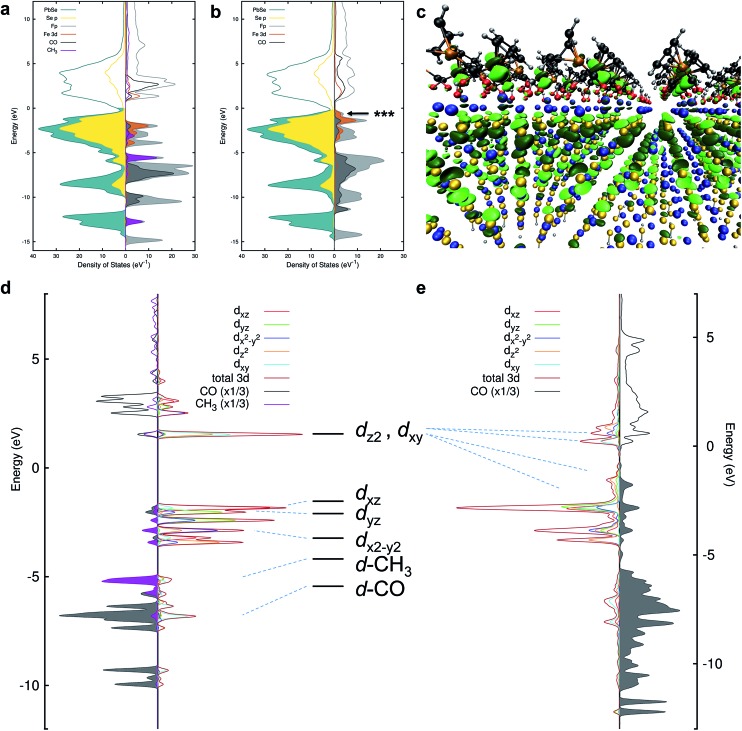
(a) Projected density of states (pDOS) for PbSe and Me-Fp in an unbound configuration. The NC states are shown to the left and selected Me-Fp states to the right. Filling of the curves indicates occupied states. The Fermi level has been shifted to 0 eV. (b) pDOS for PbSe-Fp in a bound configuration. (c) DFT-derived orbital isosurface of a single valence level when Fp beacons are bound to the Pb(ii)-rich {111} surface of PbSe. The nanocrystal is modeled as a 2D slab of 5 monolayers; the same atomic color scheme applies as in Fig. 1. The arrow in (b) indicates the energy of the orbital depicted by the isosurface. (d) The final-state (with an Fe 2p core-hole) pDOS of Me-Fp showing the CO and methyl pDOS to the left and the orbital-resolved Fe 3d pDOS to the right. Filled-curves for the CO and methyl pDOS indicate occupied levels; occupied Fe levels are presented as unfilled curves for the purpose of clarity. The Fermi level is shifted to 0 eV. (e) The final-state pDOS for PbSe-Fp. The level diagram appearing between (d) and (e) is the canonical diagram for 3d levels in a *C*_4v_ symmetry environment. The dashed lines indicate the approximate correspondence to the DFT-calculated pDOS.

The spectroscopic sensitivity of the HMB's d-orbitals allows us to interrogate the nature of their bonding to the NC surface. Fe L-edge X-ray emission spectroscopy (XES) was performed on PbSe-Fp films and compared to a methylated small molecule analogue (Me-Fp) to probe whether Fp beacons forge bonds to PbSe surfaces through their occupied 3d-orbitals. The electronic transition from occupied 3d → 2p core hole showed very similar structure for both PbSe-Fp and Me-Fp, however the transition for PbSe-Fp occurred 0.6 eV lower in energy (Fig. 3a). This shift to lower binding energy for PbSe-Fp is consistent with our hypothesis that Fp forms bonds to the PbSe surface and that binding is concomitant with effective orbital mixing across the PbSe-Fp interface, which lowers the energies of occupied Fe 3d states relative to vacuum. This orbital mixing (Fig. 4a and b) is evident in DFT-derived isosurface plots of the valence levels in which NC states show clear 3d-character at Fe (Fig. 4c). The coupling of Mp d-levels and NC valence states is precisely why they can serve as HMBs, both to modify and probe spectroscopically the surface electronic structure of the NCs with high specificity.

### Assessment of the re-organization of electron density across HMB-nanocrystal interfaces

The nature of 3d-orbital mixing and charge re-organization at the PbSe-Fp interface was further characterized with Fe L-edge X-ray absorption spectroscopy (XAS) and DFT. Bonding between Fe and its two CO ligands involves two synergistic interactions: (1) σ-donation from filled CO σ-orbitals to empty Fe 3d-orbitals, and (2) π back-donation from filled Fe 3d-orbitals to empty π*-orbitals on CO ligands.^45^–^47^ Complexes of the type L–Fp, where L represents an ancillary ligand coordinated to the Fe center, have been shown to dramatically re-distribute electron density about Fe by perturbing the extent to which Fe can engage in π back-donation with CO ligands.^48^ For Me-Fp, the methyl group serves as a good σ-donor and negligible π-acceptor, and thus significant π back-donation from Fe to CO is observed. Where PbSe has been re-surfaced with Fp beacons, it is now appropriate (albeit atypical) to consider the PbSe surface as the ancillary ligand L to Fe.

We found Fe L-edge XAS particularly useful as a direct measure of Fe π-back bonding^49^ to CO for both Me-Fp and PbSe-bound Fp beacons; Fig. 3b shows the Fe L_2,3_-edge XAS spectra for Me-Fp and PbSe-Fp, which probes a nominal Fe 2p-3d transition. The L_3_ edge for PbSe-Fp has one main absorption feature at 706.3 eV, whereas Me-Fp has two with approximately equal intensity at 707.9 eV and 709.9 eV. While the final-state unoccupied density of states is not a true proxy for L-edge XAS, qualitative understanding can still be gained from considering the level diagrams for the empty states. Complexes incorporating the Fp moiety have pseudo-octahedral symmetry.^49^–^52^ Here the Fe site of the Fp molecule has *C*_4v_ symmetry, leading to a low-spin configuration with occupied 3d_*xz*_, 3d_*yz*_, and 3d_*x*^2^–*y*^2^_ Fe orbitals. The 3d_*z*^2^_ and 3d_*xy*_ orbitals appear at higher energy and are unoccupied.^51^ This atomic level-splitting scheme is well reproduced by our DFT-generated ground-state density of states for Me-Fp (not shown) and generally preserved in the presence of an Fe 2p core-hole (Fig. 4d). From the Me-Fp final-state projected DOS in Fig. 4d, there are two degenerate lowest-energy empty Fe states, which involve hybridization of the 3d_*z*^2^_ orbital with the methyl substituent and the 3d_*xy*_ orbital with the CO ligands. Transitions into these two states give the peak at 707.9 eV. At higher energy, there is a group of states with mixed d-orbital character, and transitions into these levels yield the second L_3_ peak at 709.9 eV.

When the Fp ligand is bound to the PbSe surface, there is significant mixing of the orbital character of the Fe 3d-levels as they hybridize strongly with the NC states, principally of Se 4p character. The energies of the Fe states adjust to the energy bands of the NC (see Fig. 4b); particularly, the unoccupied Fe levels shift down in energy to align with the NC conduction band. The lowest-energy unoccupied Fe levels of Me-Fp, having 3d_*z*^2^_ and 3d_*xy*_ character, are redistributed to various states leaving only a single group of unoccupied Fe states (Fig. 4e). Thus, the Fe L-edge XAS of PbSe-Fp shows only one main feature, rather than the two seen for Me-Fp, and the energy of this transition is lower than both peaks in the Me-Fp spectrum. Pinning the energy levels of the Fp molecule to the NC band structure lowers some of the unoccupied states of the molecule for Fp bound to PbSe; this leads to a charge transfer of approximately half an electron from the PbSe NC to each bound Fp molecule.

Leveraging the sensitivity of C=O vibrational frequency to the iron center's bonding configuration, FT-IR analysis was obtained for these complexes. The CO symmetric and asymmetric stretches for PbSe-bound Fp beacons with local *C*_s_ symmetry were present at higher wavenumbers (*ν*_CO_ = 2033 and 1991 cm^–1^) than those for Me-Fp (*ν*_CO_ = 2007 and 1944 cm^–1^) (Fig. S9†). While the charge reorganization evident by L-edge XAS suggests that PbSe transfers charge to the Fp complex, which would lower *v*_CO_, the observed increase in the energy of the CO vibration relative to Me-Fp suggests severe steric crowding.

We anticipate that binding efficacy of Mp beacons to different semiconductor NC surfaces will depend critically on the extent to which charge reorganization at the metal center can be exploited to form strong heterometallic bonds, in addition to steric constraints.

### Unusual structural perturbations at the nanocrystal surface imposed by HMBs

**Fig. 5:**
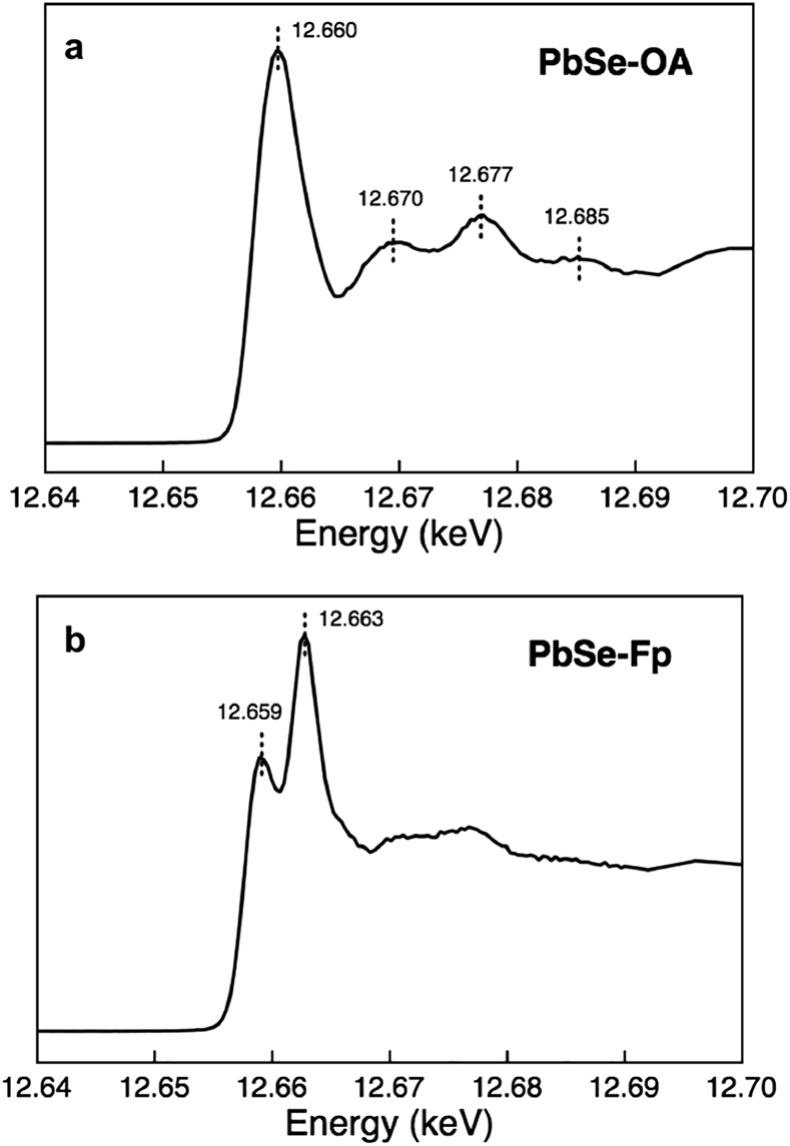
Se XAS evidencing significant surface distortions in PbSe-Fp. (a) Se K-edge XAS of PbSe-OA. (b) Se K-edge XAS of PbSe-Fp.

**Fig. 6:**
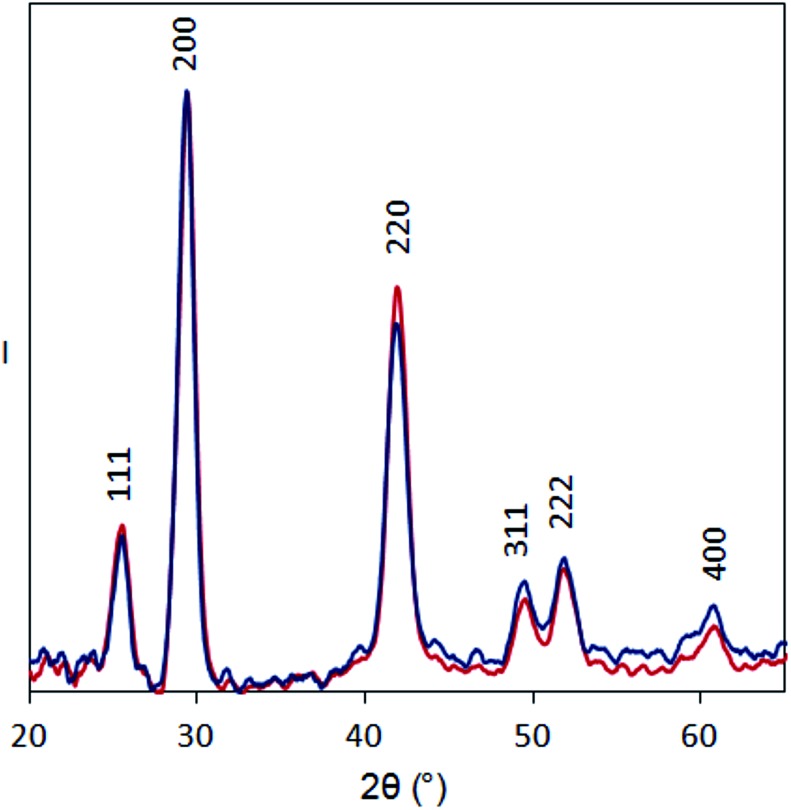
XRD patterns for PbSe-OA (blue) and PbSe-Fp (red) films on glass substrates. Intensity is normalized to the 200 peak to facilitate comparison.

**Fig. 7:**
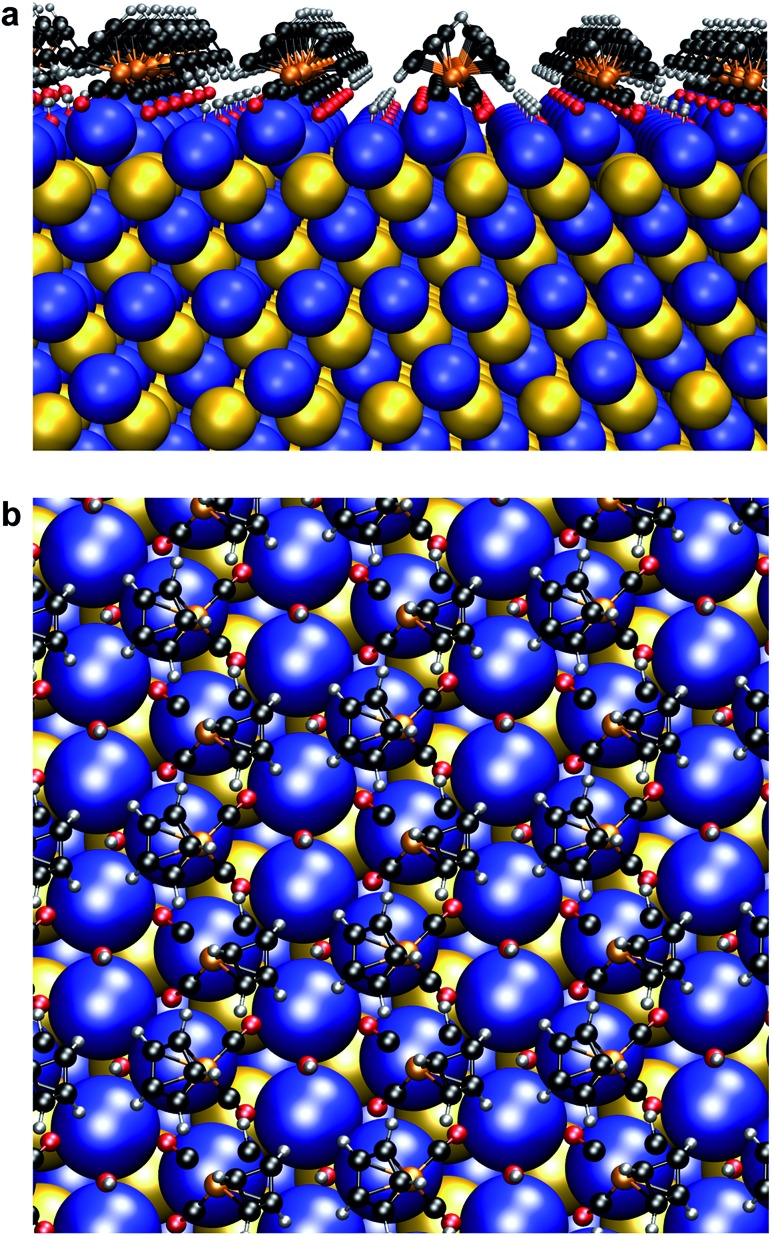
Surface atomic structure of Fp beacons on the {111} face of PbSe. (a) Side-on view. (b) Top-down view. Following the work of Zherebetskyy *et al.*, one molar equivalent of –OH ligands relative to surface Pb(ii) and Fp were used for charge balance.^55^

We found that Se K-edge XAS, which probes the unoccupied states of Se with primarily 4p character, provided exquisite sensitivity to changes in surface composition, local coordination geometry of surface Se, and local electronic environment for core Se due to charge screening by HMBs present at the surface. For PbSe-OA, a sharp, single absorption feature at 12.660 keV for the 1s → 4p transition was observed; the extended region's well-defined oscillations indicated high uniformity of local atomic structure about Se sites throughout the NC (Fig. 5a). The spectrum was quantitatively similar to bulk rock-salt PbSe. For PbSe-Fp on the other hand, the Se K-edge XAS exhibited two peaks not observed in either bulk PbSe or PbSe-OA: one at 12.659 keV, and the other at 12.663 keV (Fig. 5b). We interpret the lower energy peak at 12.659 keV as arising from the collective influence of strong Fp donors at the NC surface on the electronic environment experienced by core Se (*i.e.*, due to charge screening). This is consistent with our experimental and theoretical descriptions of through-bond coupling and electronic charge re-organization at NC-HMB interfaces (Fig. 3). For the higher energy feature at 12.663 keV, we find evidence of tetrahedral distortions in the coordination environment of surface Se. This assignment was made after comparison with related observations made for sphalerite CdSe (tetrahedral Se environments) of different sizes and organic capping ligands,^53^ and noting that Fp binding to PbSe does not change the NC core's rock-salt lattice (Fig. 6). This interpretation is further supported by the Se K-edge XAS data for PbSe-Fp in the extended region (Fig. 5b), which showed broader, less defined structure than was observed for PbSe-OA (Fig. 5a). We also noted in our DFT calculations of the lead-rich {111} surface of PbSe-Fp that the surface structure becomes distorted (Fig. 7), in particular when surface hydroxyls are included in the simulation, whose presence was recently noted for PbS nanocrystals.^54^,^55^

We suspect that atomic re-organization as observed here is a general outcome for interfacing species with strict stereoelectronic constraints—including chalcogenidometallates,^56^–^58^ polyoxometalates,^59^,^60^ and possibly perovskites.^61^ Careful delineation of those outcomes likely deserves special consideration in the future by methods outlined here and elsewhere.

## Conclusions

We have shown that the interplay of semiconductor composition and d-block heterometallic surface constituents presents a versatile paradigm to understand surface structure and bonding in colloidal semiconductor nanocrystals. By appending these unusual molecular beacons to nanocrystal surfaces through heterometallic bonds, we are able to couple the NC states to d-orbitals at the transition element. We anticipate that in future schemes, heterometals with different coordination spheres and electronic/spin configurations will become critical tools to manipulate the electronic structure of NCs, inaccessible using organic or main-group ligands. These new schemes can be deliberately tailored to direct the flow of electrons, spin and energy through NC active layers. Photo-, thermal- and electro-chemical activation of NC-bound heterometallics will provide an additional mechanism for modulating properties. It should therefore be possible to build, from this fundamental understanding of NC surface–ligand interactions established by our work, a predictive framework guiding the use of heterometals at semiconductor NC surfaces to achieve desirable properties for better photo(electro)catalytic, thermoelectric, spintronic, topological insulator, photovoltaic and FET devices.

## Supplementary Material

Supplementary information
