# Supplementary material for: Chemically directing d-block heterometallics to nanocrystal surfaces as molecular beacons of surface structure
Source: Chem Sci. 2015 Jul 28;6(11):6295–304. doi: 10.1039/c5sc01474c (PMC6054122; doi:10.1039/c5sc01474c)
Supplement: Supplementary file 1 [file SC-006-C5SC01474C-s001.pdf]

Electronic Supplementary Material (ESI) for Chemical Science.
This journal is © The Royal Society of Chemistry 2015

Electronic Supplementary Information

for

Chemically Directing *d*-Block Heterometallics to Nanocrystal Surfaces as Molecular Beacons of Surface Structure

Evelyn L. Rosen,[a] Keith Gilmore,[a] April M. Sawvel,[a] Aaron T. Hammack,[a] Sean E. Doris,[a,b] Shaul Aloni,[a] Virginia Altoe,[a] Dennis Nordlund,[c] Tsu-Chien Weng,[c] Dimosthenis Sokaras,[c] Bruce E. Cohen,[a] Jeffrey J. Urban,[a] D. Frank Ogletree,[a] Delia J. Milliron,[a,d] David Prendergast,[a] and Brett A. Helms[*,a]

[a] The Molecular Foundry, Lawrence Berkeley National Laboratory, Berkeley, CA 94720 United States.

[b] Department of Chemistry, University of California, Berkeley, CA 94720 United States.

[c] Stanford Synchrotron Radiation Lightsource, SLAC National Accelerator Laboratory, Menlo Park, CA 94025 United States.

[d] McKetta Department of Chemical Engineering, The University of Texas at Austin, Austin, Texas 78712, United States.

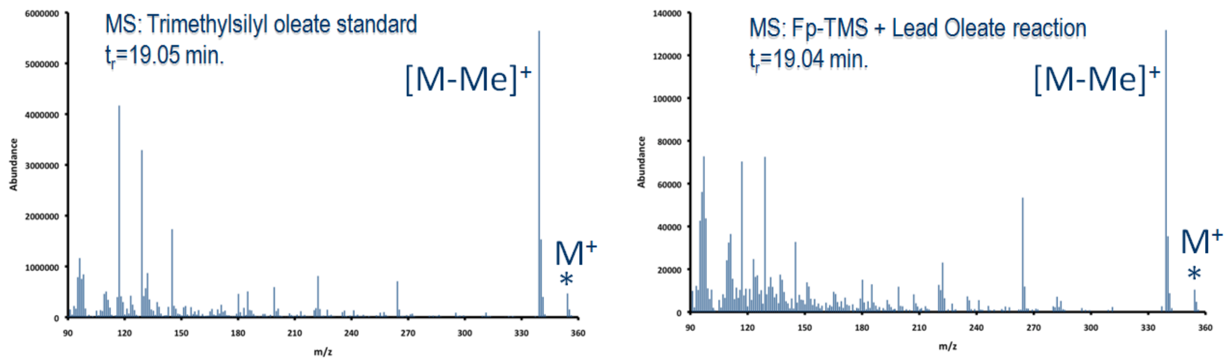

Figure S1. GC-MS of trace of trimethylsilyl oleate (*left*) and the reaction of $Pb(OA)_2$ with Fp-TMS (*right*). The formation of trimethylsilyl oleate in this model system supports chemically directed ligand exchange for oleate-passivated PbSe NCs.

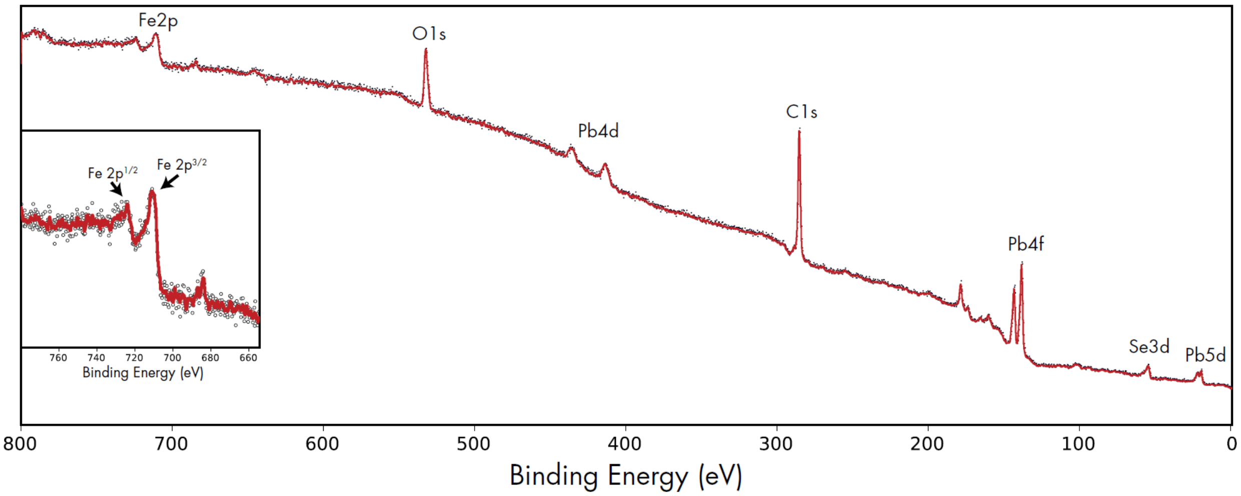

Figure S2. XPS for a PbSe-Fp film on Si. Iron, lead, carbon, and oxygen peaks are evident, matching the expected ligand and nanocrystal composition. The inset shows the measured iron $2p^{1/2}$ and $2p^{3/2}$ peaks at 723 eV and 711 eV, respectively, which supports the successful substitution of oleate ligands by Fp molecular beacons at the PbSe NC surface. In both the full spectrum and inset, the black points correspond to the raw data, while the red line shows the results of a Savitzky-Golay smoothing filter (6 points, 4th order) applied to the data.

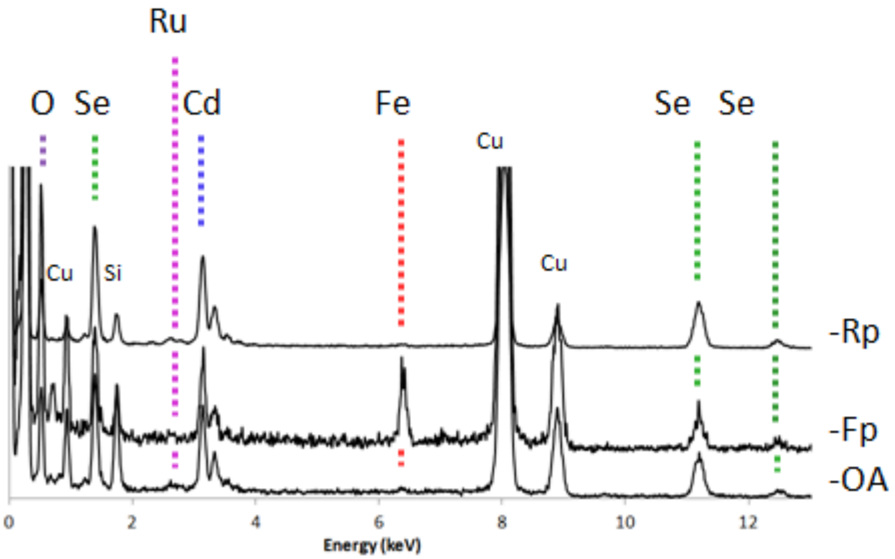

Figure S3. EDS of CdSe-OA, CdSe-Fp, and CdSe-Rp nanocrystals on a carbon film TEM grid.

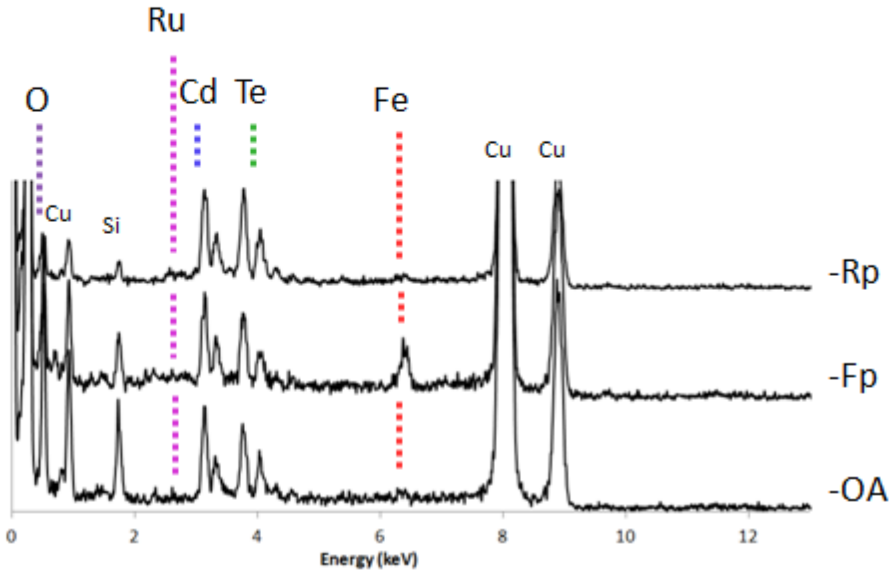

Figure S4. EDS of CdTe-OA, CdTe-Fp, and CdTe-Rp nanocrystals on a carbon film TEM grid.

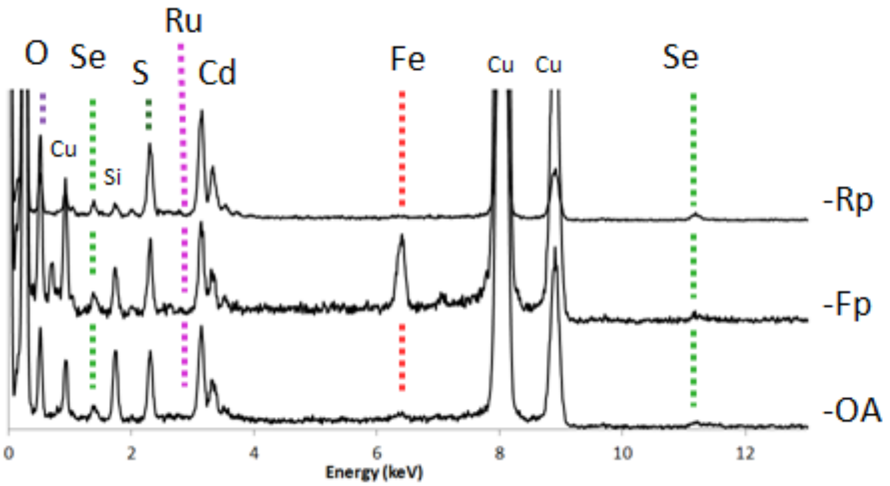

Figure S5. EDS of CdSe/CdS-OA, CdSe/CdS-Fp, and CdSe/CdS-Rp nanocrystals on a carbon film TEM grid.

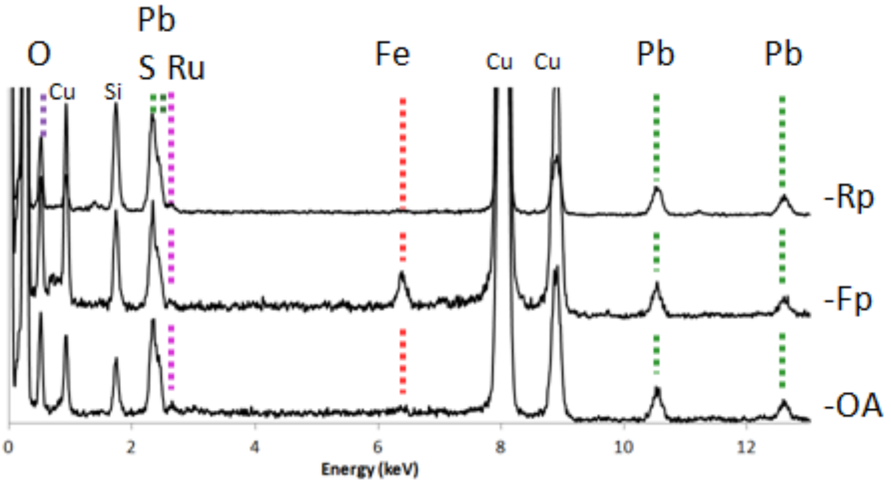

Figure S6. EDS of PbS-OA, PbS-Fp, and PbS-Rp nanocrystals on a carbon film TEM grid.

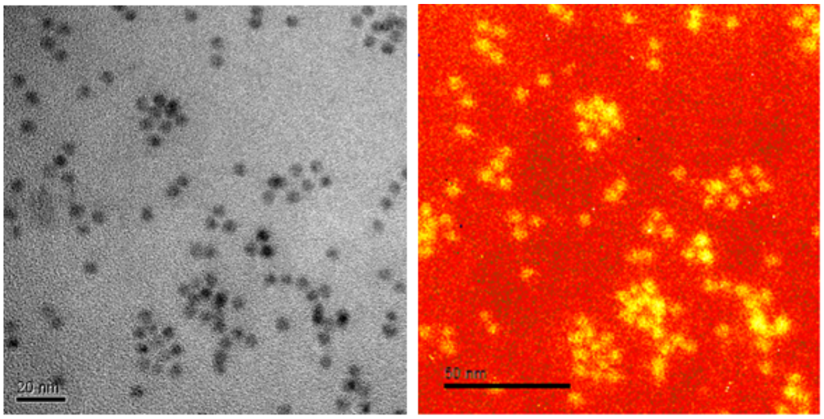

Figure S7. Bright field view for CdSe-Fp nanocrystals on a carbon film TEM grid (*left*) and corresponding Fe-$L_{2,3}$ map (*right*).

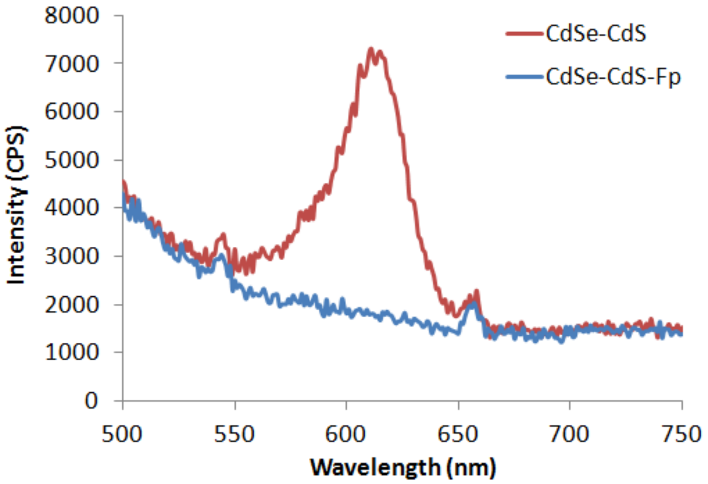

Figure S8. Photoluminescence spectra for CdSe-CdS (*red*) and CdSe-CdS-Fp (*blue*) films.

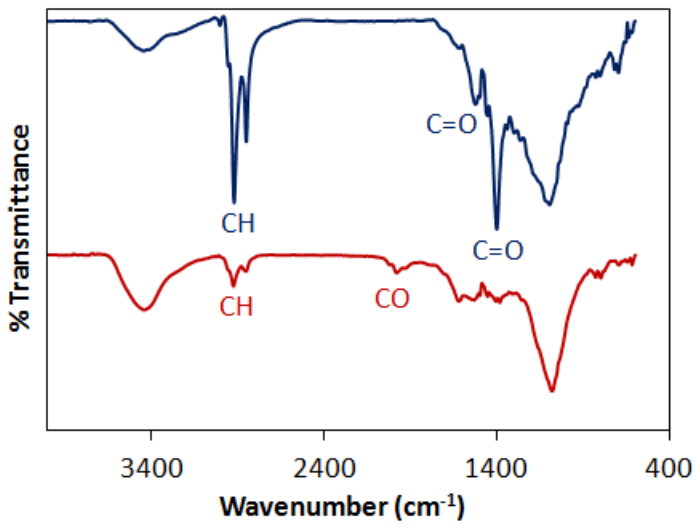

Figure S9. FT-IR of PbSe-OA (*blue*) and PbSe-Fp (*red*) in KBr. Spectra have been vertically offset for clarity.

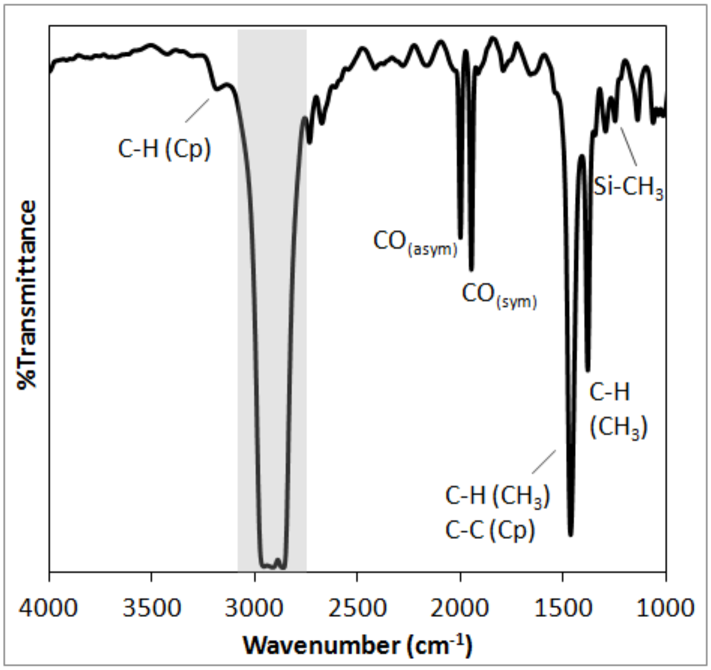

Figure S10. FT-IR of TMS-Fp under inert atmosphere (CaF_2 windows) in hexanes. The shaded region is attributed to hexanes absorption.

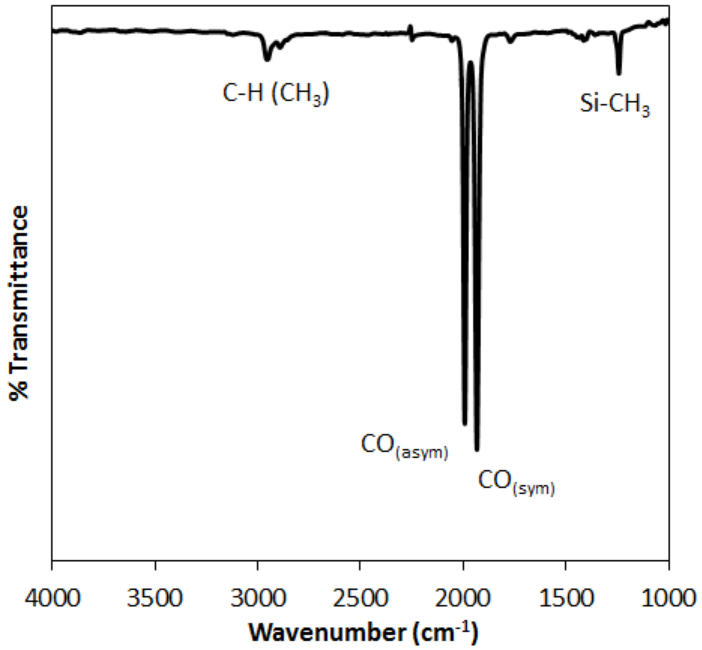

Figure S11. FT-IR of TMS-Fp under inert atmosphere (CaF_2 windows) in $CDCl_3$.

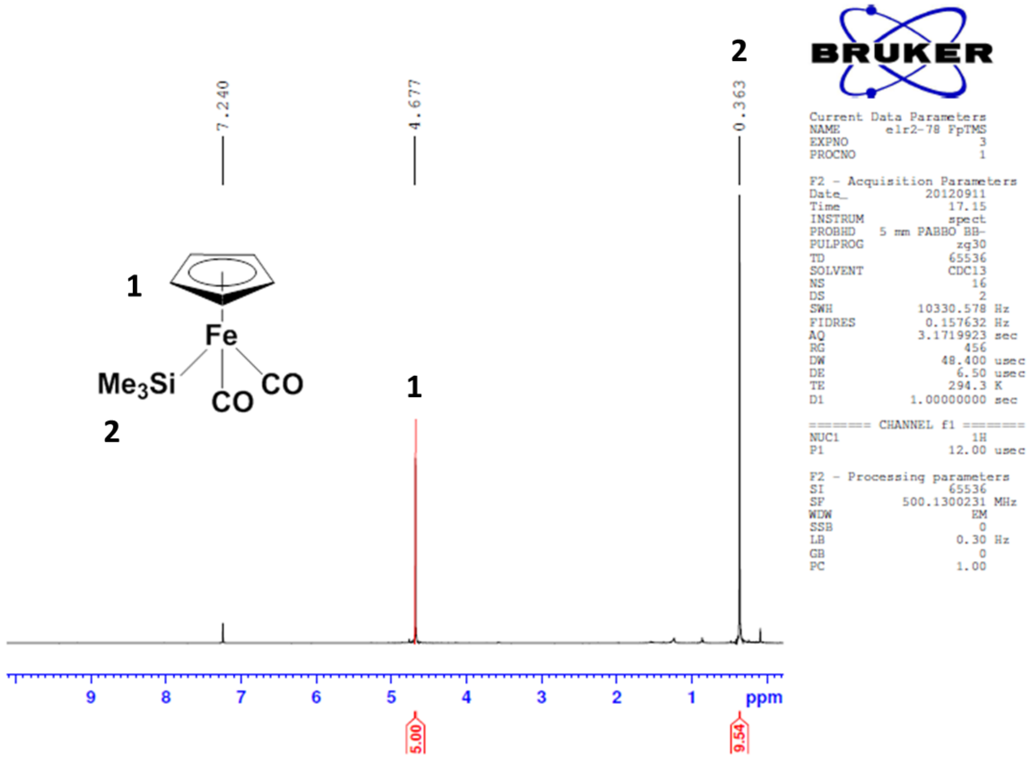

Figure S12. ^1H NMR spectrum for TMS-Fp in $CDCl_3$ solvent.

Table S1. ICP-OES for various nanocrystal compositions. The NC samples were digested in concentrated nitric acid (>99.99 trace metals grade) and a minimum of three independently prepared samples were analyzed for each surface composition. The number of excess surface metal atoms ($M_{surface}$) and total number of metal atoms (M_{total}) were estimated using the non-stoichiometric model developed by Yu and coworkers[48].

Composition	M_{total} : Se : Mp	$M_{surface}$ (Total Atoms)	Mp : $M_{surface}$	Mp per NC	Mp/NC area (Mp/nm^2)
PbSe-OA	1.23 : 1.00 : 0.00	634 (6148)	---	---	---
PbSe-Fp	1.21 : 1.00 : 0.20	579 (6093)	0.95 : 1.00	550	3.2
PbSe-Rp	1.22 : 1.00 : 0.10	607 (6121)	0.43 : 1.00	260	1.5
CdSe-OA	1.11–1.23 : 1.00 : 0.00	29–61 (559–591)	---	---	---
CdSe-Fp	1.09 : 1.00 : 0.17	24 (554)	1.82 : 1.00	43	1.1
CdSe-Rp	1.13 : 1.00 : 0.06	34 (564)	0.46 : 1.00	16	0.4
